# Supplementary material for: Telomere length of both parents contributes to heritable POT1 cancer-predisposition syndrome
Source: Nat Commun. 2026 Sep 14;17:10797. doi: 10.1038/s41467-026-77414-2 (PMC13669393; doi:10.1038/s41467-026-77414-2)
Supplement: Supplementary file 1 [file 41467_2026_77414_MOESM1_ESM.pdf]

Supplementary Information

Supplementary Table 1: heterozygous POT1 mutation profiles of assayed patient samples

Family	Relationship	POT1 Status	Samples Source	Age at the time of samples collection
1	Father (F1)	POT1+ (c.1851_1852del;p.Asp617GlufsTer9)	peripheral blood	43
1	Mother (M1)	-	peripheral blood	40
1	Proband (first sample, P1(a))	POT1+ (c.1851_1852del;p.Asp617GlufsTer9)	peripheral blood	5
1	Proband (second sample, P1(b))	POT1+ (c.1851_1852del;p.Asp617GlufsTer9)	peripheral blood	6
2	Father (F2)	POT1+ (c.107T>C;p.Tyr36His)	peripheral blood	57
2	Mother (M2)	-	peripheral blood	58
2	Proband (P2)	POT1+ (c.107T>C;p.Tyr36His)	peripheral blood	26
2	Cousin (C2)	-	peripheral blood	30
3	Mother M3	POT1+ (c.265_273delin AATCTT;p.Tyr89_Lys91delinsAsnLeu)	peripheral blood	67
3	Proband (first sample, P3(a))	POT1+ (c.265_273delin AATCTT;p.Tyr89_Lys91delinsAsnLeu)	bone marrow	12
3	Proband (second sample, P3(b))	POT1+ (c.265_273delin AATCTT;p.Tyr89_Lys91delinsAsnLeu)	bone marrow	17
3	Proband (second sample, P3(c))	POT1+ (c.265_273delin AATCTT;p.Tyr89_Lys91delinsAsnLeu)	bone marrow	17
3	Sister (S3)	POT1+ (c.265_273delinsAATCTT;p.Tyr89_Lys91delinsAsnLeu)	peripheral blood	33
3	Husband (H3)	-	peripheral blood	41
3	Nephew (N3.1)	-	peripheral blood	15
3	Nephew (N3.2)	-	peripheral blood	15
3	Nephew (N3.3)	POT1+ (c.265_273delinsAATCTT;p.Tyr89_Lys91delinsAsnLeu)	peripheral blood	13
3	Nephew (N3.4)	-	peripheral blood	11
3	Nephew (N3.5)	POT1+ (c.265_273delinsAATCTT;p.Tyr89_Lys91delinsAsnLeu)	peripheral blood	9
4	Proband (P4)	POT1+ c.1087C>T;p.Arg363Ter	peripheral blood	5
5	Proband (P5)	POT1+ c.1071dup;p.Gln358SerfsTer13	peripheral blood	14
N/A	Control (Ctrl A)	-	peripheral blood	56
N/A	Control (Ctrl B)	-	peripheral blood	48
N/A	Control (Ctrl C)	-	peripheral blood	38

Supplementary Table 2: Telobait sequence for oligo synthesis ordering (5' - 3')

Note the phosphorylation and 3'-Biotin-TEG modification for Telobait_GO_top. Telobait_GO_bot1-6 sequences are not modified. All Telobait sequences were ordered from IDT with HPLC purification.

**Telobait _top**	/Phos/AAGGTTAACACAAAGACACCGACAACTTTCTTCAGCACCTGATATCACTTTTAC/3BioTEG/
**Telobait_bot1**	GTAAAAGTGATATCAGGTGCTGAAGAAAGTTGTCGGTGTCTTTGTGTTAACCTTCTAACCCTA
**Telobait_bot2**	GTAAAAGTGATATCAGGTGCTGAAGAAAGTTGTCGGTGTCTTTGTGTTAACCTTTAACCCTAA
**Telobait_bot3**	GTAAAAGTGATATCAGGTGCTGAAGAAAGTTGTCGGTGTCTTTGTGTTAACCTTAACCCTAAC
**Telobait_bot4**	GTAAAAGTGATATCAGGTGCTGAAGAAAGTTGTCGGTGTCTTTGTGTTAACCTTACCCTAACC
**Telobait_bot5**	GTAAAAGTGATATCAGGTGCTGAAGAAAGTTGTCGGTGTCTTTGTGTTAACCTTCCCTAACCC
**Telobait_bot6**	GTAAAAGTGATATCAGGTGCTGAAGAAAGTTGTCGGTGTCTTTGTGTTAACCTTCCTAACCCT

Supplementary Table 3: list of TVR sequence abbreviations and plot color used for Telogator2 analysis

Category	Sequence	Symbol	Plot Color (Python Colorname)
Canonical	TTAGGG	C	lightgrey
C-type	TCAGGG	D	gold
	TCAGG, TCACG, …	E	seagreen
G-type	TGAGGG	F	indianred
	TGAGG, TGACGG	G	navy
J-type	TTGGGG	H	yellowgreen
	TGGGGG, TTTGGG	I	royalblue
Common Variations	CGAGGG	K	darkseagreen
	CTAGGG	L	cornflowerblue
	CTAGGGG, CTAGG	M	deepskyblue
	TTCGGG	N	goldenrod
	TCGGG, TCGGGG, …	P	darkorange
Rare Variations	CGGGGG, CCAGGG, …	Q	steelblue
	CTGGGG	R	rebeccapurple
	TTA	S	mediumvioletred
Canonical Indels	TTAAGGG	T	darkmagenta
	TAGGGG, TTAGGGG, …	V	teal
Errors	TAGG	W	firebrick

For a full list of all k-mers annotated, see Telogator2 documentation.

Supplementary Figures

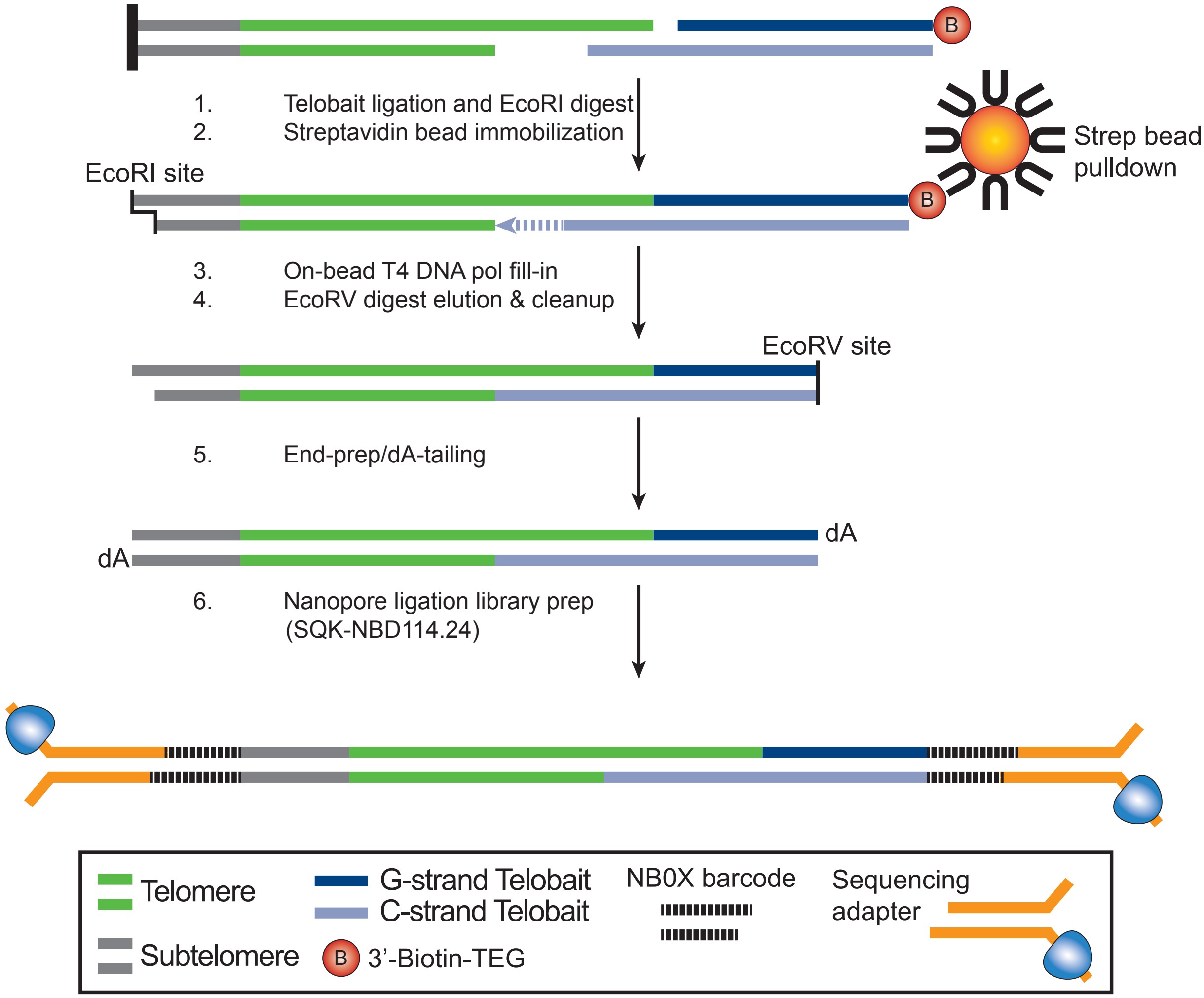

Supplementary Figure 1: Schematic of telomere enrichment and nanopore library preparation

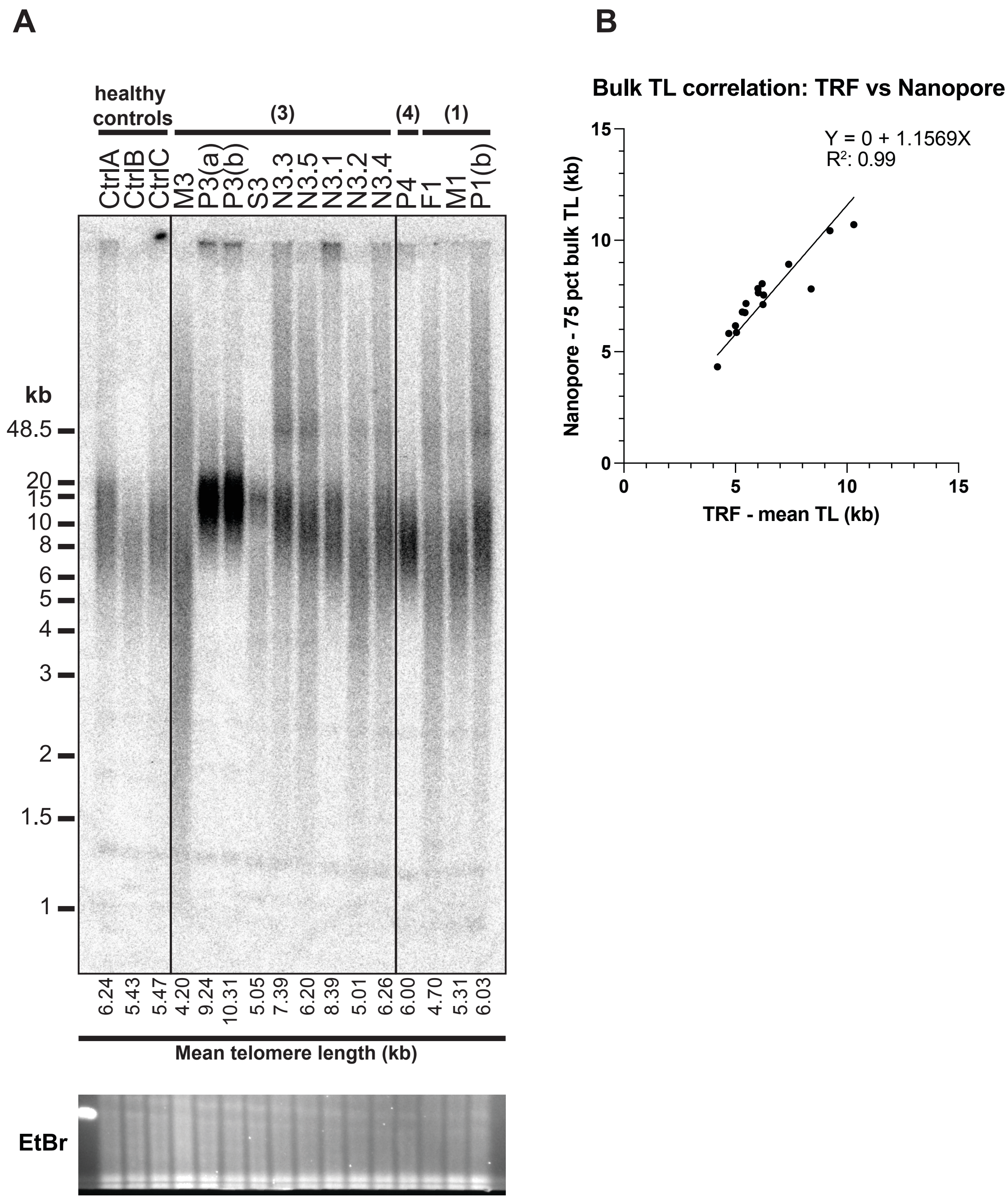

Supplementary Figure 2: Comparison of telomere restriction fragment length analysis to nanopore sequencing

(A) Telomere restriction fragment (TRF) length analysis of n=16 healthy unrelated controls, POT1 patient DNA samples used in this study from Family (1), (3), (4) as shown in Figure 1A. Mean telomere length determined by densitometry analysis of TRF are indicated at the bottom of each lane alongside ethidium bromide (EtBr) staining. (B) Linear regression analysis of mean TRF telomere length measurements from (A) and corresponding 75 pct telomere length of the same n=16 samples analyzed via nanopore

sequencing. Telomere length from Nanopore sequencing is ATL (adjusted telomere length), where ATL = TL_len+tvr_len as shown in Figure 1A. TRF length calculations were performed in Excel, see Supplementary Data 2 and linear regression analysis performed using Graphpad PRISM (see Source Data - Fig1b_SuppFig2.zip). Source data provided as a source data file.

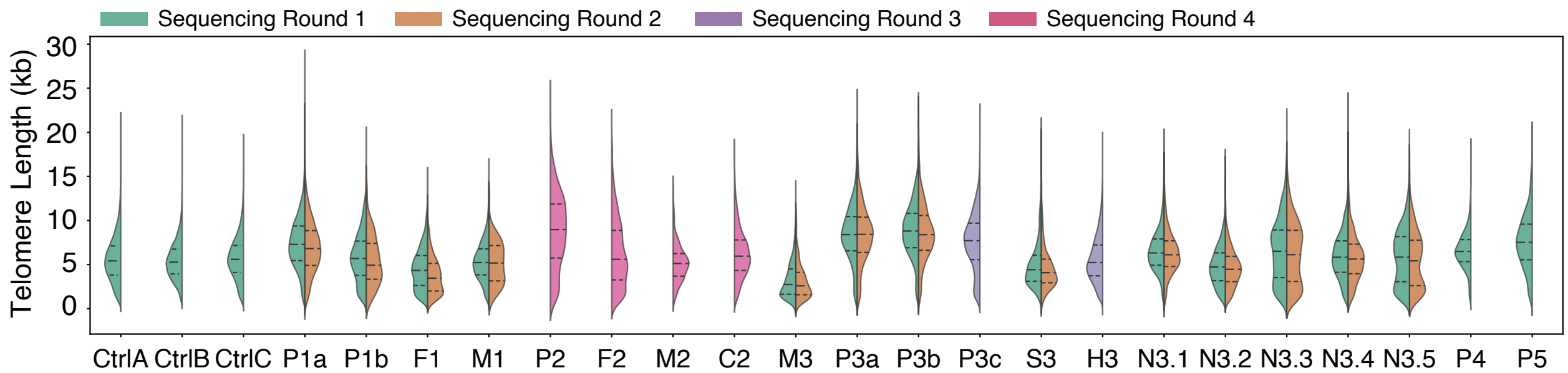

Supplementary Figure 3: Comparison of sample consistency between sequencing rounds

Bulk telomere length measurements for all samples split by sequencing round. Each sequencing round refers to independent library preparation and subsequent sequencing from the same starting DNA sample. Median and interquartile range are indicated. Source data provided as a source data file.

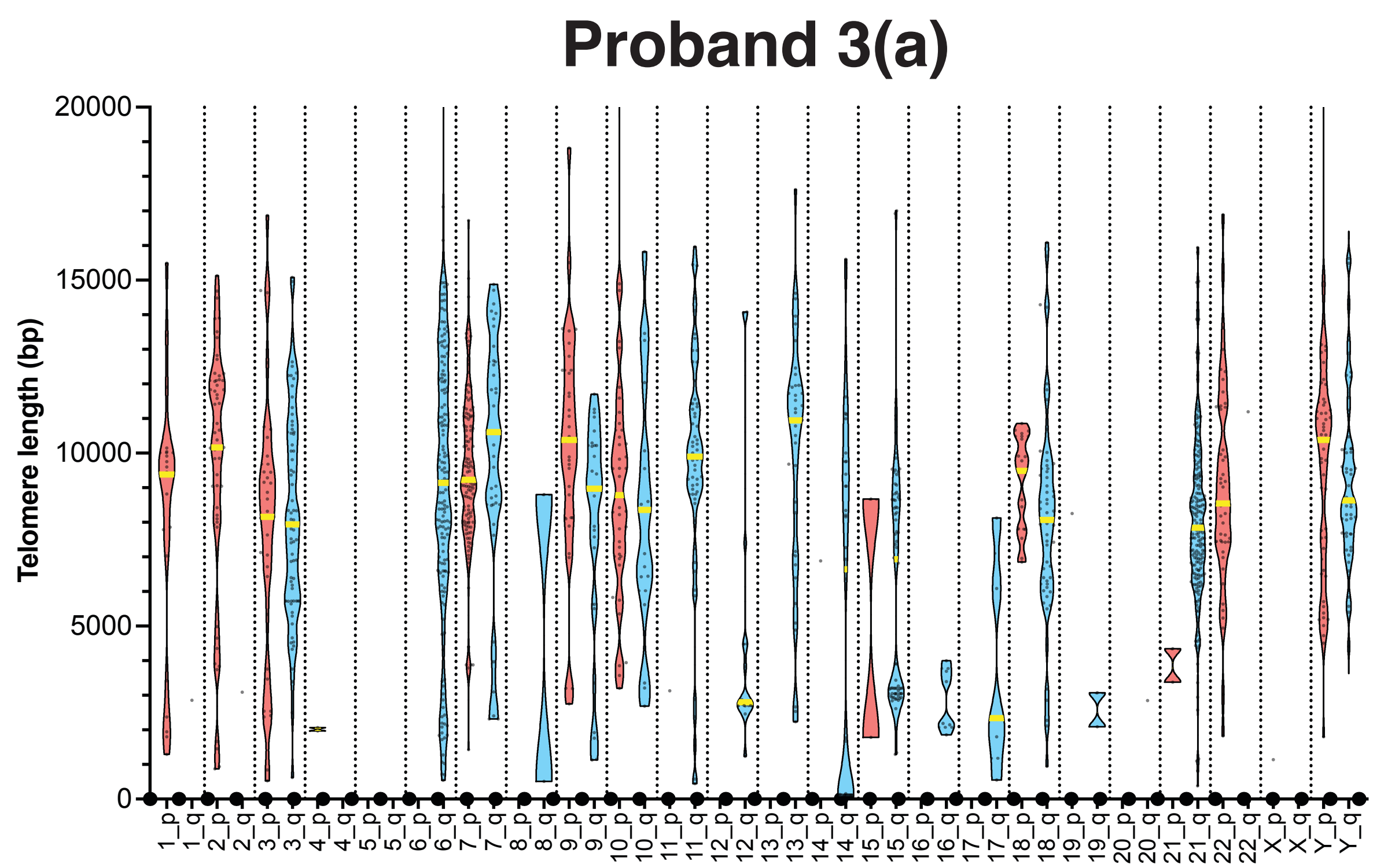
Proband 3(a)
Telomere length (bp)
20000
15000
10000
5000
0
1_p 1_q 2_p 2_q 3_p 3_q 4_p 4_q 5_p 5_q 6_p 6_q 7_p 7_q 8_p 8_q 9_p 9_q 10_p 10_q 11_p 11_q 12_p 12_q 13_p 13_q 14_p 14_q 15_p 15_q 16_p 16_q 17_p 17_q 18_p 18_q 19_p 19_q 20_p 20_q 21_p 21_q 22_p 22_q X_p X_q Y_p Y_q

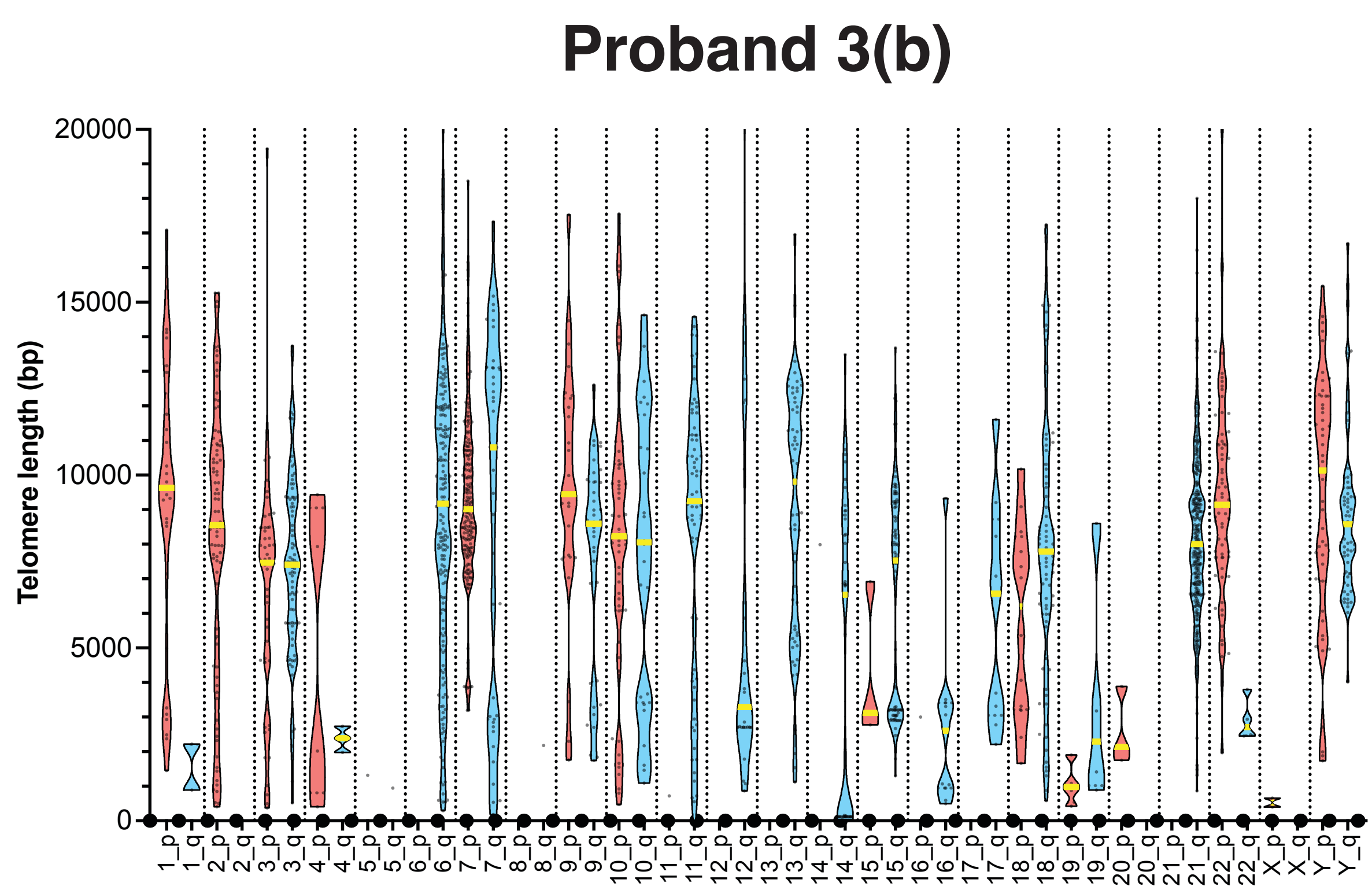
Proband 3(b)
Telomere length (bp)
20000
15000
10000
5000
0
1_p 1_q 2_p 2_q 3_p 3_q 4_p 4_q 5_p 5_q 6_p 6_q 7_p 7_q 8_p 8_q 9_p 9_q 10_p 10_q 11_p 11_q 12_p 12_q 13_p 13_q 14_p 14_q 15_p 15_q 16_p 16_q 17_p 17_q 18_p 18_q 19_p 19_q 20_p 20_q 21_p 21_q 22_p 22_q X_p X_q Y_p Y_q

Supplementary Figure 4: Chromosome-end specific telomere alignment to standard reference genome using Telometer

Chromosome-end specific telomere length measurements for samples Proband 3a (top panel), n=1961 telomeres and Proband 3b (bottom panel), n=1242 telomeres, with p-arm alignment indicated in red and q-arm alignment shown in blue using telomere[1]. Sample median displayed for each aligned sample. Due to the high heterogeneity of subtelomeric regions and loss of distal unique sequences during enrichment, on average, fewer than 61% of telomeric reads mapped confidently to unique chromosome ends with a MAPQ > 30 filter. Moreover, the distribution of aligned reads was skewed, with up to 430-fold more reads mapping to the most represented telomere compared to the least. These findings are consistent with results from alternative pipelines, underscoring the inherent challenges of chromosome-end assignment without a sample-specific telomere-to-telomere reference genome[2–5]. Moreover, chromosome-end specificity could in principle identify individual telomere pairs, but it does not resolve maternal vs. paternal inheritance or the distinct dynamics of allelic telomeres. Source data provided as a source data file.

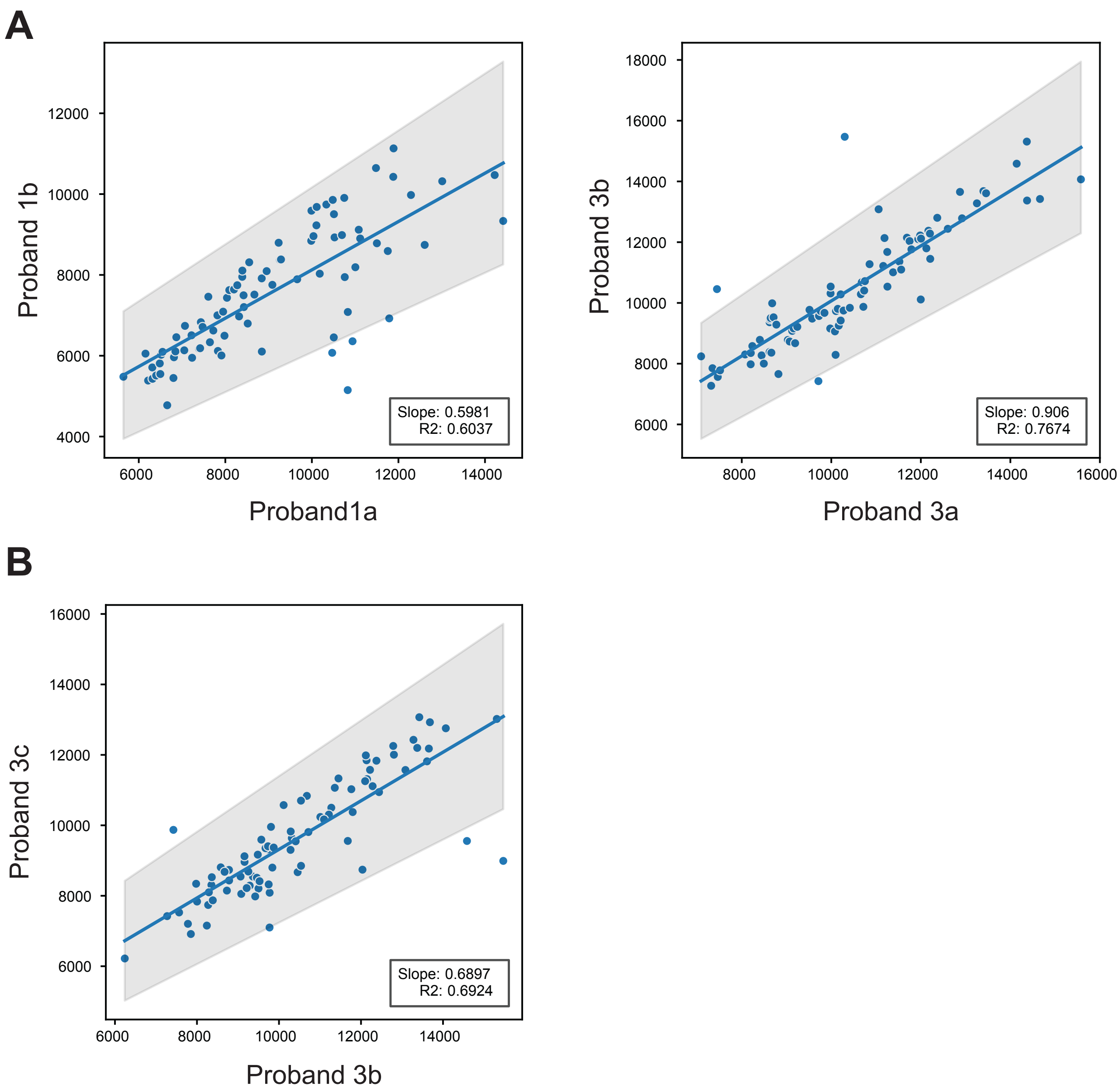

Supplementary Figure 5: Correlation of cluster-specific telomere length between separate Nanopore runs

(A) 75th percentile telomere length for each TVR cluster identified in both independently collected samples for Proband 1 and Proband 3. (B) Correlation of cluster-specific telomere length for each TVR cluster for independent sequencing runs on the same DNA sample for Proband 3. Ordinary least squares linear regression was performed using the statsmodels.api OLS module. The overall significance of the regression model was evaluated using the F-statistic p-value (p-val). Shaded region indicates the confidence interval of the linear regression. Source data provided as a source data file.

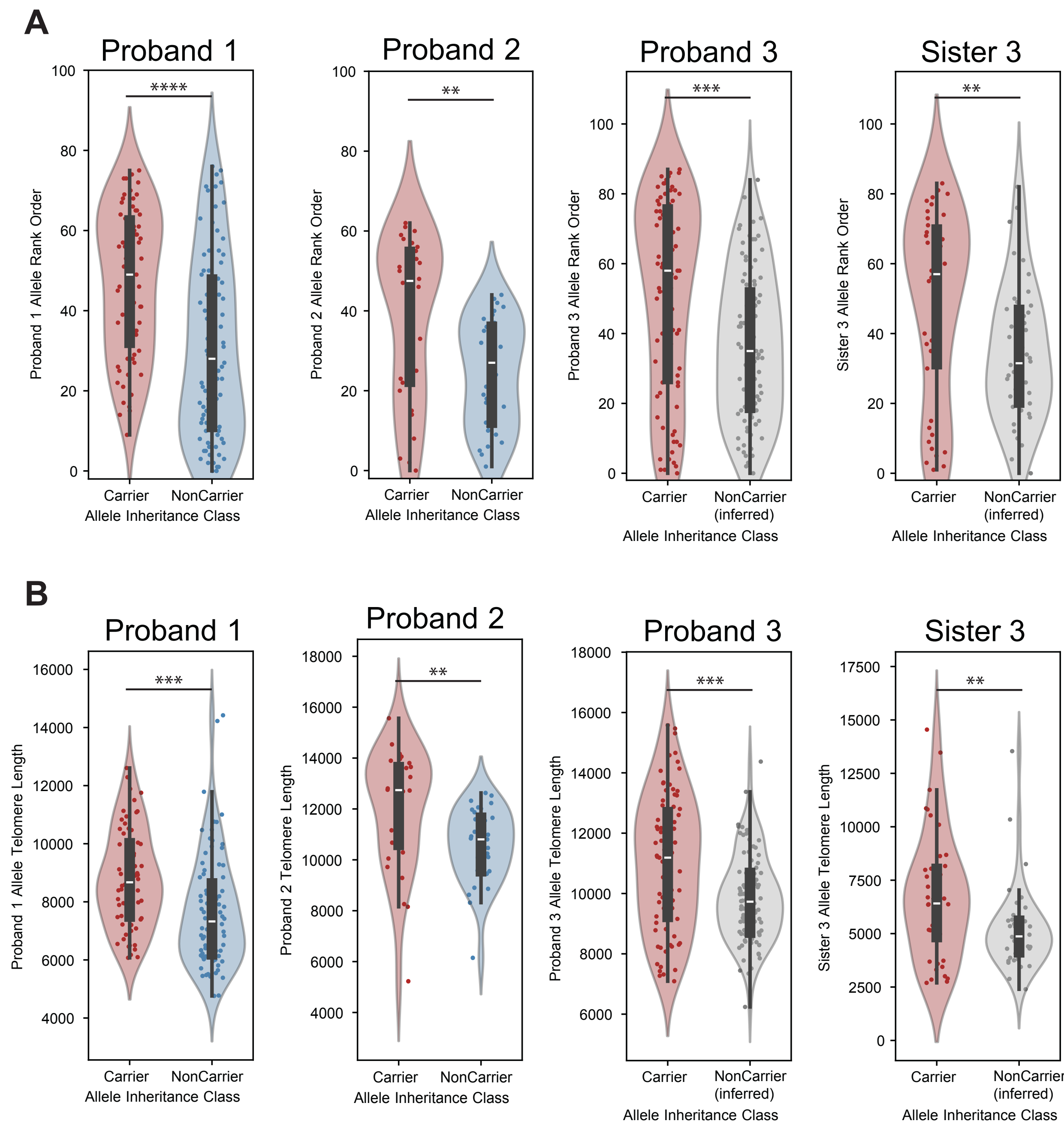

Supplementary Figure 6: Significant enrichment of carrier-inherited alleles among the longest telomeres

Intra-sample telomere rank order (A) and 75th percentile telomere length (B) plotted for each telomere allele, split by inheritance from the carrier (red) or non-carrier (blue, grey if non-carrier presumed but no non-carrier parental sample available) for the following samples: Proband 1 (left), Proband 2 (middle-left), Proband 3 (middle-right) and Sister 3 (right). Sample comparison performed using two-tailed Welch's t-test (** = p-value<0.01, *** = p-value<0.001,**** = p-value<0.0001). Exact p-values: A) Proband1: 3.0e-06,

Proband 2: 0.0020, Proband 3: 0.00019, Sister 3: 0.0090; B) Proband1: 0.00021, Proband 2: 0.0059, Proband 3: 4.5e-05 ,Sister 3: 0.0065. Source data provided as a source data file.

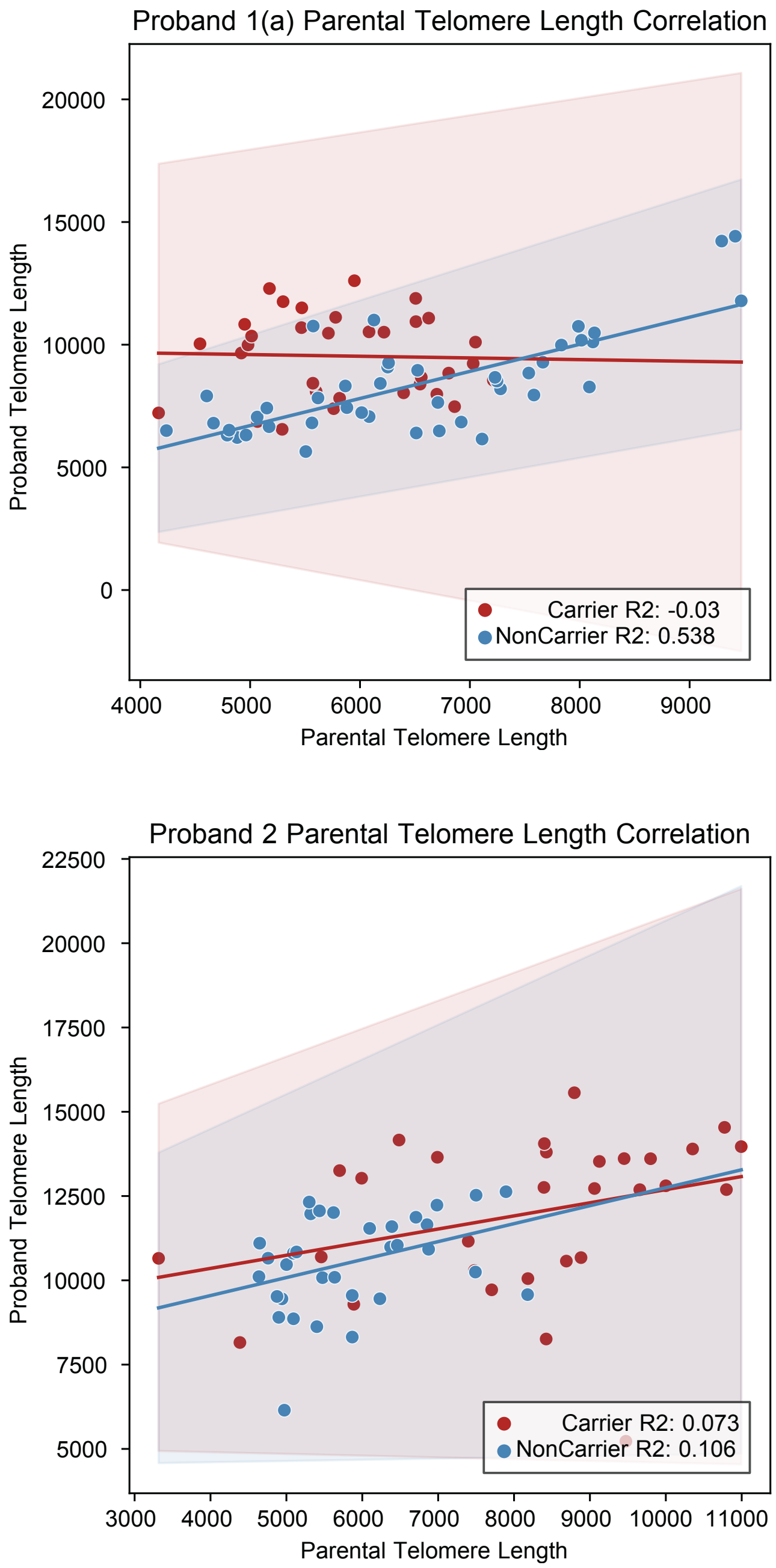

Supplementary Figure 7: Correlation of Proband and parental absolute telomere length

Proband (x-axis) and parental (y-axis) 75th percentile telomere length for shared alleles inherited from the carrier (red) and non-carrier (blue) parents for Proband 1 (A) and Proband 2 (B). Ordinary least squares linear regression was performed independently for carrier and non-carrier alleles using the statsmodels.api OLS module. The overall significance of the regression model was evaluated using the F-statistic p-value (p-val). Shaded region indicates the confidence interval of the linear regression. Source data provided as a source data file.

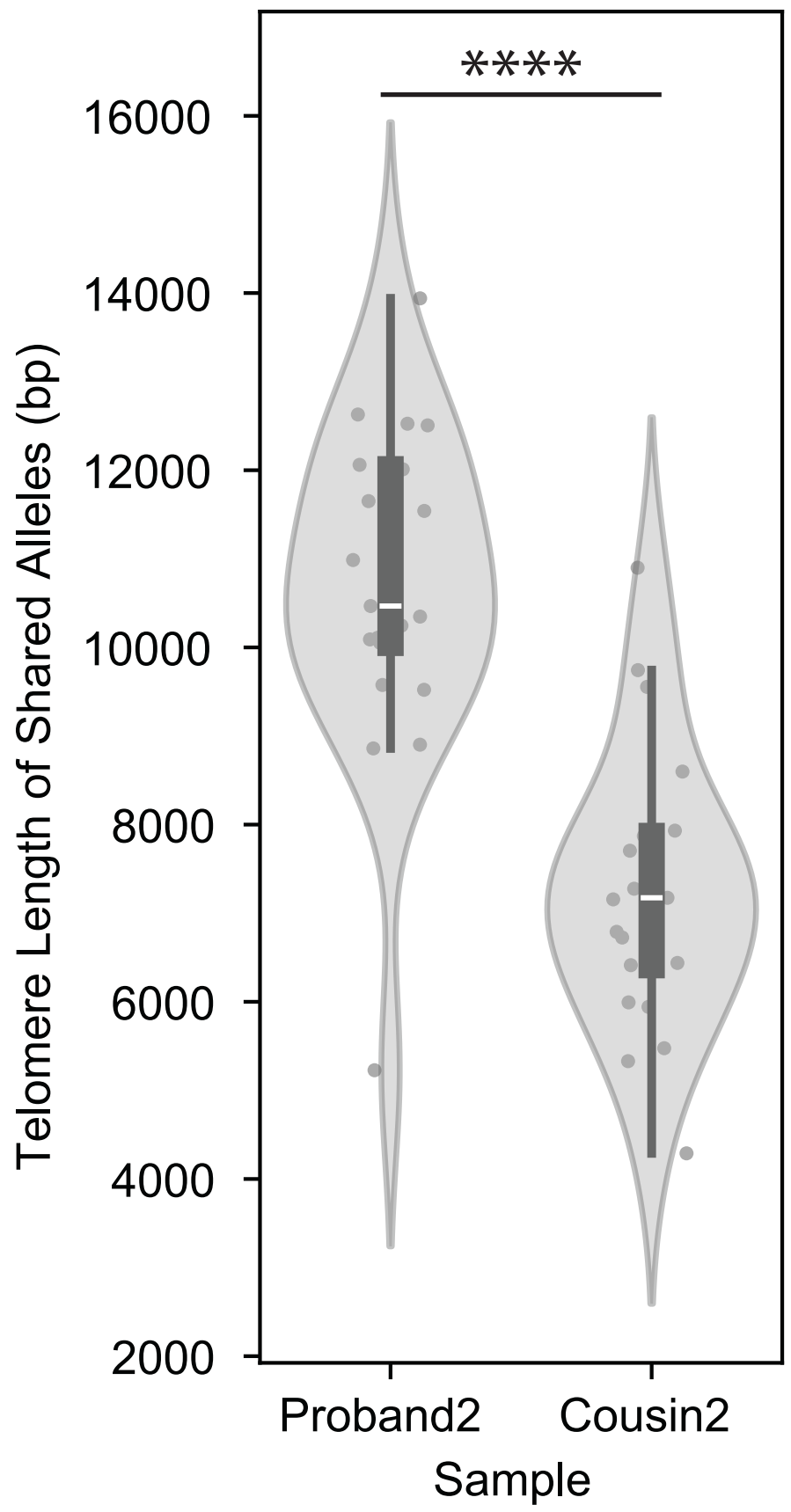

Supplementary Figure 8: Proband 2 shows significantly longer shared telomere length than their non-carrier Cousin 2

Comparison of 75th percentile telomere length between Proband 2 and Cousin 2, subset to include only shared TVR haplotypes. Sample comparison performed using two-tailed Welch's t-test (**** = p-value 9.9e-08). Source data provided as a source data file.

References for Supplementary Information

1. Sanchez, S. E. *et al.* Digital telomere measurement by long-read sequencing distinguishes healthy aging from disease. *Nat. Commun.* **15**, 5148 (2024).
2. Smoom, R. *et al.* Telomouse—a mouse model with human-length telomeres generated by a single amino acid change in RTEL1. *Nat. Commun.* **14**, 6708 (2023).
3. Karimian, K. *et al.* Human telomere length is chromosome end-specific and conserved across individuals. *Science* **384**, 533–539 (2024).
4. Schmidt, T. T. *et al.* High resolution long-read telomere sequencing reveals dynamic mechanisms in aging and cancer. https://doi.org/10.1101/2023.11.28.569082 (2023) doi:10.1101/2023.11.28.569082.
5. Smoom, R., Lichtental, D., Kaestner, K. H. & Tzfati, Y. The house mouse maintains constant telomere length throughout life. *Nucleic Acids Res.* **53**, gkaf830 (2025).
