# Supplementary material for: Telomere length of both parents contributes to heritable POT1 cancer-predisposition syndrome
Source: Nat Commun. 2026 Sep 14;17:10797. doi: 10.1038/s41467-026-77414-2 (PMC13669393; doi:10.1038/s41467-026-77414-2)
Supplement: Supplementary file 2 [file 41467_2026_77414_MOESM2_ESM.pdf]

nature portfolio

Peer Review File

Telomere length of both parents contributes to heritable POT1 cancer-predisposition syndrome

Corresponding Author: Dr Dirk Hockemeyer

This file contains all reviewer reports in order by version, followed by all author rebuttals in order by version.

Version 0:

Reviewer comments:

Reviewer #1

(Remarks to the Author)
In this manuscript the Hockemeyer lab is taking advantage of a novel nanopore long read telomere sequencing approach to decipher the inheritance of telomere length in POT1 cancer families. Telomere length was markedly increased on average, with a fraction of the telomeres rendered over 10kb by the POT1 mutations. Haplotype specific markers for chromosome ends, which could only be resolved by the new sequencing approach, allowed the tracing of telomere inheritance independently of family specific reference genomes. This approach revealed that long telomeres were preferentially inherited from the mutation carrier, and short telomeres from the non-carrier. However rank order comparison also revealed that the long telomeres inherited from the carrier were not fully reset during embryogenesis, while short telomeres maintained their anti-proliferative potential. To understand the mechanism the group compared carrier to non-carrier siblings, which revealed that unaffected individuals showed a clear distinction between inherited long (from carrier parents) and short (from non-carrier parents) telomeres. Fascinatingly, this was very different in affected offspring, where POT1 mutations strongly favored elongation of the shorter telomeres. The approach uncovered a novel mechanism for genetic anticipation, where the presence of long telomeres directs how short telomeres are elongated, depending on POT1 status.
This is truly an amazing manuscript, as it contains many firsts:
- The recently developed ONT sequencing approaches for telomeres, coupled with the haplotype analysis here, opens new ways to understand telomeropathies. The Hockemeyer group recognized this and applied the approaches to POT1 mutations.
- Clearly, we did not understand how telomere length differences are inherited. The results here reveal that our hypothesis of telomere length being reset during embryogenesis were rather naïve.
- The discovery of the mechanism of how long inherited telomeres can bias how short ones are elongated by telomerase dependent on the POT1 mutation context, suggests much tighter crosstalk between telomeres than anticipated.
- Understanding the mechanisms of how even non-carrier offspring of POT1 mutant parents are cancer prone has deep therapeutic importance.
In summary, this manuscript and the discoveries it contains are extremely important. The impact on the telomere field and community is obvious, but it carries much further than that, as the discoveries contain a novel telomere driven genetic anticipation mechanism that potentially predisposes non carriers to cancer. The approaches contained in the manuscript are technically challenging but carried out impeccably. As such, the manuscript is perfectly suitable for Nature Communications (honestly, given the novelty and results, I would have preferred to review this for Nature).
Minor:
Please fix the first sentence in the second to last paragraph of the results section.
The discussion could benefit from more emphasis and speculation of how the signaling from short telomeres, including their inflammatory phenotype, is suppressed through the presence of long telomeres. This is a major discovery and only modestly outlined.

Reviewer #2

(Remarks to the Author)
The study conducted by A. Martin et al. investigates the hypothesis of a correlation between specific germline POT1 variants that determine telomere hyperelongation and an anticipation effect in offspring of carrier parents, while also evaluating the mechanistic contribution from the non-carrier parent.

The methodology is innovative, consistent, and thoroughly described.

I will not reiterate the limitations already acknowledged by the authors, including the limited sample size and the absence of trio analysis for the second family.

However, I have some suggestions/comments to better contextualize the strengths and weaknesses of the results.

1. The investigated hypothesis appears to rely on a single reference (Ref. 4) and therefore appears inherently weak. Additional supporting evidence would strengthen the proposed mechanistic model. This is only a suggestion. I acknowledge the preliminary nature and innovative nature of the data presented, at a time when only recent studies are beginning to differentiate the distinct functional impact of germline POT1 variants (e.g. deepscan mutagenesis studies).

2. While the clinical histories are suggestive, direct evidence demonstrating causality or correlation with the described germline variants is lacking. Some diagnoses, based on their type or age at onset, may also not be attributable to these variants. The clinical spectrum of POT1-associated syndromes remains a subject of ongoing debate (see UK Guidelines), and the assumed correlation warrants further validation, and maybe a comment.

3. Nevertheless, the conclusion is focused on proposing a mechanistic model describing telomere length dynamics in relatives of POT1 variant carriers, without addressing the clinical spectrum of the syndrome. However, I would suggest using a less assertive term than "establish", as other variants (with different impacts on POT1 function and structure, cell viability, and damage response), may differentially affect telomere length regulation/reset during the zygotic phase.

In summary, I consider that the study has weaknesses, perhaps also due to its innovative nature, but the refined methodological approach could serve as a framework to further explore the dynamics of telomere elongation, and their contribution to a molecular environment favorable to tumorigenesis.

Reviewer #3

(Remarks to the Author)
In the present study, Martinez and colleagues analyzed telomere length by nanopore sequencing in individuals from two families carrying POT1 mutations associated with long telomeres, as well as two isolated POT1 mutation carriers. Some mutations in POT1 have been demonstrated to induce aberrant telomere elongation and are associated with an increased predisposition to carcinogenesis, a condition that has recently been termed "long telomere syndrome."
Long-read sequencing of telomeres allows for the distinction of parental origin of each telomere from all chromosomes. The results indicate that long telomeres can be transmitted through inheritance, and that telomere elongation post-fertilization promoted by POT1 mutation has a predilection for affecting the short telomeres from the non-carrier parent's chromosomes. The work is derived from expert groups in telomere biology and POT1 which have been among the firsts to publish the technique that allows for the sequencing of telomere via Nanopore. While the question of whether and how long telomeres are passed on to offspring in families carrying POT1 mutations is of great interest, the evidence provided in this study is insufficient to answer the question precisely.

Major concerns :
- The authors have previously reported the methodology for analyzing telomere length by ONT sequencing (Sanchez et al., Nat Commun 2024). In this article, the capture of the telomeric ends in conjunction with restriction digestion of genomic DNA resulted in the enrichment of telomeric reads by several thousand-fold, as compared to whole genome sequencing. Nevertheless, the present study by Martin et al., demonstrates that the enrichment is merely 44 times. It is imperative that the authors provide an explanation for this suboptimal efficiency in telomeric enrichment. This has a restrictive effect on the extent to which the chromosomal and haplotype-specific telomere length analysis can be conducted for each sample. This low enrichment has been demonstrated to have a significant impact on the number of telomeres that can be sequenced per chromosome and haplotype, thereby reducing the depth of analysis of telomere diversity and heterogeneity.

- As outlined by the authors themselves at the conclusion of the discussion, the primary issue and limitations of this article are as follows. Due to the absence of certain samples (Fathers of P2 and S2 ; Father of N2.1, N2.2, N2.3, N2.4 and N2.5), a comprehensive analysis of the two families cannot be conducted. Further families with POT1 mutations and age-matched controls would be required to consolidate the observations.

- The three controls used are adults (the youngest is 38 years old, whereas most individuals tested who carry POT1 mutations are younger). Since, as demonstrated by the authors in a previous article using the same technique (Sanchez et al., Nat Comm 2024), telomere length is highly dependent on age, age-matched controls would be necessary.

- The authors write « On average, probands displayed median telomere lengths ~1.9 kb longer than our three controls (age 56, 48, and 38)… ; ». Nevertheless, this observation is not pertinent, given that the probands P1 and P2 were analyzed at ages 5/6 and 12/17, respectively, whereas the control group comprised individuals between the ages of 38 and 56. This comparison is meaningless.

- Samples P2a and P2b are derived from bone marrow, while all other samples analyzed in this study are from blood. It is important to note that the authors have previously demonstrated that the telomere length in bone marrow cells was

significantly higher than that of blood cells in the same individual (Fig3a in Sanchez et al., Nat Comm 2024). Therefore, it can be concluded that including these two samples in the comparative study would not be rigorous. P2 underwent a bone marrow transplant, which explains why blood is not available, but comparing bone marrow and blood samples from different individuals does not seem appropriate to me.

- The authors write « 7 of 11 POT1 carriers had a significantly enriched fraction of long telomeres (>10 kb) compared to controls ». However, there are 9 POT1 carriers analyzed, not 11. Duplicate from the same individual cannot be considered as independent POT1 carriers.

- Again, 7 of the 9 POT1 carriers are below age 34 while the youngest control is 38. Age-matched controls are necessary to reliably analyze interindividual variation of telomere length.

Minor :
- To stenghen this study, it would be interesting to test the telomere length of individuals' sperm (where possible). This could help to verify that « the germline elongation « scrambles » the original telomere length order in the carrier » as proposed by the authors.

- Blood cells carrying POT1 mutations are prone to clonal hematopoiesis (DeBoy et al., NEJM 2023), a phenomenon that could affect telomere homeostasis. It would therefore be interesting to extend the analysis of telomeres in tissues other than blood in carriers of the POT1 mutation, such as fibroblasts or epithelial cells obtained from nasal brushing (a minimally invasive method of obtaining cells).

- It is written that « Proband samples from Families 1 and 2 were sequenced twice from independent clinical samples collected 5 years apart » however in Suppl Table 1. P1a and P1b samples are collected at age 5 and 6, respectively.

- Figure 2C : The authors state that the TVR is shared by six samples. However, the profiles of samples P2a and P2b appear to differ from those of the other samples. How would the authors explain this?

- The telomere length in P1b is strongly reduced as compare to P1a while there is only 1 year of difference between the two samples. A previous study has showed that POT1 carriers have a slower rate or telomere shortening that healthy donors and non-carriers in blood cells over time (DeBoy et al., NEJM 2023). How the authors can explain this difference between the two samples P1a and P1b?

- How to explain that M2 and S2 who are both POT1 mutation carriers have so short telomeres?

- The analysis of telomere length in P4 is not relevant as there are not age-matched controls.

- The authors mention that germline mutations in POT1 are associated with familial cancer predisposition and long telomeres. However, to be exhaustive, the authors should also mention that germline mutations in POT1 have also been identified in telomeropathies with short telomeres (Tummala et al. EMBO mol med, 2024 ; Kelich, et al. J. Exp. Med 2022) as well as in Coat plus syndrome with long telomeres (Takai et al. Genes and Dev. 2016).

- The ages indicated in brackets in Figure 1 and in the Supplementary table do not correspond.

- Telomere length profiles in samples N2.3 and N2.5 appear bimodal with ONT sequencing (Fig.1B) but not with TRF (Suppl. Fig1). Could you comment?

- The sentence « Despite these dramatic differences in absolute telomere length, rank-order analysis revealed a clear heriSupplementary Table ignature, with haplotypes from the POT1 carrier mother enriched in the longest third of telomeres and depleted in the shortest third in both siblings » should be edited.

Version 1:

Reviewer comments:

Reviewer #1

(Remarks to the Author)
The authors have addressed my concerns and comments and I am fully supportive of publication.

(Remarks on code availability)

Reviewer #2

(Remarks to the Author)
I thank the authors for the work done during the revision.
The additions are substantial.

These were not formal requests of mine, and I want to acknowledge that the authors went well beyond what I asked.
The new data also do something more important than addressing my previous comments as they replicate the central observation independently, and this is precisely the kind of internal validation that a study based on so few families needs.
On my three original points I have little to add.
The wording on generational anticipation is now careful and, in my view, correct.
The authors no longer claim clinical evidence, they propose a mechanism, and they spell out the conditions under which such a mechanism would translate into observable anticipation. The same applies to the discussion on cross-family comparison and allele-specific penetrance, which I had raised as a clinical concern. The new paragraph acknowledges it explicitly.
One reference I would suggest adding is the recent UK clinical practice guidelines for POT1-TPDS (Hanson and colleagues, J Med Genet 2025). They are, to my knowledge, the only formal national guideline on this syndrome. They are also relevant to the authors' caveat, because the UK panel explicitly stepped back from a Li-Fraumeni-equivalent surveillance model, noting that current evidence does not support comparable lifetime cancer risk. Citing this work would strengthen the clinical contextualisation of the manuscript and would also signal to readers where the field currently stands.
The findings do not, at this stage, justify any change to surveillance protocols, predictive testing thresholds, or reproductive counselling for non-carrier offspring of POT1 mutation carriers.
This is consistent with the tone the authors have already adopted and would prevent the kind of over-reading that, in my experience, tends to happen with mechanistic papers in rare cancer predisposition syndromes.
With this addition I support publication.

(Remarks on code availability)
The code is a usable resource, though I did not find any readme file with enough instructions for installing and running the application

Reviewer #3

(Remarks to the Author)
I thank the authors for their careful and substantive revision of the manuscript. I have reviewed the point-by-point response, the revised manuscript, the updated supplementary materials, and the revised figures.
Overall, the manuscript is now significantly improved in clarity, balance, and interpretive rigor.
I support publication in Nature communications, pending only minor editorial modifications.

• Line 92 typo “do”

• Lines: 262-263. As all the nephews are healthy (lines 142-143) please change affected/unaffected by carrier/non-carrier in lines 262-263.
• Many figures are of very poor quality (low definition)

(Remarks on code availability)
I thank the authors for their careful and substantive revision of the manuscript. I have reviewed the point-by-point response, the revised manuscript, the updated supplementary materials, and the revised figures.
Overall, the manuscript is now significantly improved in clarity, balance, and interpretive rigor.
I support publication in Nature communications, pending only minor editorial modifications.

• Line 92 typo “do”

• Lines: 262-263. As all the nephews are healthy (lines 142-143) please change affected/unaffected by carrier/non-carrier in lines 262-263.
• Many figures are of very poor quality (low definition)

Open Access This Peer Review File is licensed under a Creative Commons Attribution 4.0 International License, which permits use, sharing, adaptation, distribution and reproduction in any medium or format, as long as you give appropriate credit to the original author(s) and the source, provide a link to the Creative Commons license, and indicate if changes were made.
In cases where reviewers are anonymous, credit should be given to 'Anonymous Referee' and the source.
The images or other third party material in this Peer Review File are included in the article's Creative Commons license, unless indicated otherwise in a credit line to the material. If material is not included in the article's Creative Commons license and your intended use is not permitted by statutory regulation or exceeds the permitted use, you will need to obtain permission directly from the copyright holder.
To view a copy of this license, visit https://creativecommons.org/licenses/by/4.0/

RESPONSE TO REVIEWERS' COMMENTS

We would like to thank each reviewer for their positive feedback and constructive suggestions. As outlined in detail below, we have revised the manuscript and addressed all comments.

In addition to the requested revisions, we have incorporated substantial new data that further strengthen the conclusions of the study. Specifically, we added an additional POT1 family, including a complete parent–child trio as well as an unaffected maternal cousin. Telomere nanopore sequencing of this newly added Family 2 provides independent support for the central conclusions of the manuscript: that POT1 mutations drive telomere elongation in the germline, resulting in preferential inheritance of long telomeres from the carrier parent in the next generation. This family also provides an additional dataset to evaluate post-fertilization telomere elongation affecting both maternal- and paternal-derived telomeres in POT1 mutation carriers.

Moreover, we successfully obtained and sequenced DNA from the father of the five nephews in Family 3 (previously Family 2). Inclusion of this sample now allows unambiguous assignment of maternal and paternal haplotypes in the nephews and enables direct quantification of telomere elongation across parental chromosome ends in carrier and non-carrier siblings. These analyses demonstrate that the shortest telomeres undergo preferential elongation in POT1 mutation carriers, providing additional evidence for a potential mechanism by which progressive depletion of critically short telomeres inherited from the carrier parent could impair telomere-based tumor suppression across generations.

Reviewer #1 (Remarks to the Author): expertise in telomere biology and POT1

We would like to thank the reviewers for their insightful comments and constructive suggestions. In response, we have revised the manuscript to better contextualize our findings and more carefully frame the interpretation of our experiments. In particular, we have reframed the Discussion to more clearly distinguish between our experimental observations and the proposed mechanistic model. We have also added a revised model figure illustrating telomere inheritance and progressive telomere elongation dynamics in POT1 mutation carriers across generations.

In this manuscript the Hockemeyer lab is taking advantage of a novel nanopore long read telomere sequencing approach to decipher the inheritance of telomere length in POT1 cancer families. Telomere length was markedly increased on average, with a fraction of the telomeres rendered over 10kb by the POT1 mutations. Haplotype specific markers for chromosome ends, which could only be resolved by the new sequencing approach, allowed the tracing of telomere inheritance independently of family specific reference genomes. This approach revealed that long telomeres were preferentially inherited from the mutation carrier, and short telomeres from the non-carrier. However rank order comparison also revealed that the long telomeres inherited from the carrier were not fully reset during embryogenesis, while short telomeres maintained their anti-proliferative potential. To understand the mechanism the group compared carrier to non-carrier siblings, which revealed that unaffected individuals showed a clear distinction between inherited long (from carrier parents) and short (from non-carrier parents) telomeres. Fascinatingly, this was very different in affected offspring, where POT1 mutations strongly favored elongation of the shorter telomeres. The approach uncovered a novel mechanism for genetic anticipation, where the presence of long telomeres directs how short telomeres are elongated, depending on POT1 status.

This is truly an amazing manuscript, as it contains many firsts:

- The recently developed ONT sequencing approaches for telomeres, coupled with the haplotype analysis here, opens new ways to understand telomeropathies. The Hockemeyer group recognized this and applied the approaches to POT1 mutations.

- Clearly, we did not understand how telomere length differences are inherited. The results here reveal that our hypothesis of telomere length being reset during embryogenesis were rather naïve.
- The discovery of the mechanism of how long inherited telomeres can bias how short ones are elongated by telomerase dependent on the POT1 mutation context, suggests much tighter crosstalk between telomeres than anticipated.
- Understanding the mechanisms of how even non-carrier offspring of POT1 mutant parents are cancer prone has deep therapeutic importance.

In summary, this manuscript and the discoveries it contains are extremely important. The impact on the telomere field and community is obvious, but it carries much further than that, as the discoveries contain a novel telomere driven genetic anticipation mechanism that potentially predisposes non carriers to cancer. The approaches contained in the manuscript are technically challenging but carried out impeccably. As such, the manuscript is perfectly suitable for Nature Communications (honestly, given the novelty and results, I would have preferred to review this for Nature).

Minor:
Please fix the first sentence in the second to last paragraph of the results section.

We have made adjustments to this paragraph as suggested.

The discussion could benefit from more emphasis and speculation of how the signaling from short telomeres, including their inflammatory phenotype, is suppressed through the presence of long telomeres. This is a major discovery and only modestly outlined.

We have modified the discussion to better highlight the novelty of our work. Specifically, we have expanded our discussion and included an additional figure outlining our model for generational anticipation.

Reviewer #2 (Remarks to the Author): expertise in POT1 mutations in clinical genetics

We would like to thank the reviewer for providing a clinical perspective and greatly appreciate the suggestions.

The study conducted by A. Martin et al. investigates the hypothesis of a correlation between specific germline POT1 variants that determine telomere hyperelongation and an anticipation effect in offspring of carrier parents, while also evaluating the mechanistic contribution from the non-carrier parent.

The methodology is innovative, consistent, and thoroughly described.

I will not reiterate the limitations already acknowledged by the authors, including the limited sample size and the absence of trio analysis for the second family.

However, I have some suggestions/comments to better contextualize the strengths and weaknesses of the results.

1. The investigated hypothesis appears to rely on a single reference (Ref. 4) and therefore appears inherently weak. Additional supporting evidence would strengthen the proposed mechanistic model. This is only a suggestion. I acknowledge the preliminary nature and innovative nature of the data presented, at a time when only recent studies are beginning

to differentiate the distinct functional impact of germline POT1 variants (e.g. deepscan mutagenesis studies).

We thank the reviewer for making this important point. We now more clearly state that our experiments do not establish clinical evidence for generational anticipation and that additional data is required to support this claim. We also now provide a clearer model that explains that any generational effect is expected to be limited by changing the likelihood of short telomeres from the carrier parent contributing to tumor suppression. We agree that this important aspect was not sufficiently explained in the previous manuscript.

2. While the clinical histories are suggestive, direct evidence demonstrating causality or correlation with the described germline variants is lacking. Some diagnoses, based on their type or age at onset, may also not be attributable to these variants. The clinical spectrum of POT1-associated syndromes remains a subject of ongoing debate (see UK Guidelines), and the assumed correlation warrants further validation, and maybe a comment.

We appreciate the reviewers' concern and agree that the focus of our study was on the measurement of telomere length inheritance rather than linking individual POT1 mutations to clinical presentation. We have expanded our discussion to include this caveat, specifically stating that, "Our data further suggest that comparing telomere inheritance patterns across families carrying different POT1 mutations will likely be challenging, as the degree of germline and post-fertilization telomere elongation may depend on the penetrance of individual POT1 alleles. This is consistent with the diverse and complex tumor spectrum associated with POT1 mutation that remains a subject of ongoing debate."

3. Nevertheless, the conclusion is focused on proposing a mechanistic model describing telomere length dynamics in relatives of POT1 variant carriers, without addressing the clinical spectrum of the syndrome. However, I would suggest using a less assertive term than "establish", as other variants (with different impacts on POT1 function and structure, cell viability, and damage response), may differentially affect telomere length regulation/reset during the zygotic phase.

We agree with the reviewer and have made substantial changes to our discussion section to both clarify our proposed model and how it relates to genetic anticipation in familial cancer syndromes. Specifically, we note, "While our study was not designed to establish clinical evidence for genetic anticipation and additional supporting evidence is needed, our findings provide a potential mechanistic explanation for how variable anticipation, incomplete penetrance, and heterogeneous disease presentation could arise among carriers of the same pathogenic POT1 allele." We have further added more discussion in the inherent challenges associated with cross-family comparison: "Our data further suggest that comparing telomere inheritance patterns across families carrying different POT1 mutations will likely be challenging, as the degree of germline and post-fertilization telomere elongation may depend on the penetrance of individual POT1 alleles."

In summary, I consider that the study has weaknesses, perhaps also due to its innovative nature, but the refined methodological approach could serve as a framework to further explore the dynamics of telomere elongation, and their contribution to a molecular environment favorable to tumorigenesis.

Reviewer #3 (Remarks to the Author): expertise in POT1 mutations and clinical genetics

We appreciate the reviewers' careful consideration of our manuscript and have addressed the suggested changes as follows.

In the present study, Martinez and colleagues analyzed telomere length by nanopore sequencing in individuals from two families carrying POT1 mutations associated with long telomeres, as well as two isolated POT1 mutation carriers. Some mutations in POT1 have been demonstrated to induce aberrant telomere elongation and are associated with an increased predisposition to carcinogenesis, a condition that has recently been termed "long telomere syndrome."
Long-read sequencing of telomeres allows for the distinction of parental origin of each telomere from all chromosomes.
The results indicate that long telomeres can be transmitted through inheritance, and that telomere elongation post-fertilization promoted by POT1 mutation has a predilection for affecting the short telomeres from the non-carrier parent's chromosomes. The work is derived from expert groups in telomere biology and POT1 which have been among the firsts to publish the technique that allows for the sequencing of telomere via Nanopore. While the question of whether and how long telomeres are passed on to offspring in families carrying POT1 mutations is of great interest, the evidence provided in this study is insufficient to answer the question precisely.

Major concerns :
- The authors have previously reported the methodology for analyzing telomere length by ONT sequencing (Sanchez et al., Nat Commun 2024). In this article, the capture of the telomeric ends in conjunction with restriction digestion of genomic DNA resulted in the enrichment of telomeric reads by several thousand-fold, as compared to whole genome sequencing. Nevertheless, the present study by Martin et al., demonstrates that the enrichment is merely 44 times. It is imperative that the authors provide an explanation for this suboptimal efficiency in telomeric enrichment. This has a restrictive effect on the extent to which the chromosomal and haplotype-specific telomere length analysis can be conducted for each sample. This low enrichment has been demonstrated to have a significant impact on the number of telomeres that can be sequenced per chromosome and haplotype, thereby reducing the depth of analysis of telomere diversity and heterogeneity.

We appreciate the reviewer for pointing this out. This discrepancy primarily comes from a difference in methodology used to calculate enrichment. When we compare the percentage of telomeric reads from the initial publication (10-90 percentile range between 0.2-5%) we find that our new data falls within this expected range (0.38% of total reads with the new samples included).

It should be noted that there are also differences between the genomic DNA sample type used. Our 2024 publication uses high-concentration, freshly extracted genomic DNA from cell lines, whereas this paper relies on lower-concentration, pre-extracted genomic DNA from bio-banked patient samples. This necessitated increasing Telobait ligation volumes to 300 µl (all reaction components scaled proportionally). Downstream steps after initial Telobait ligation remain unchanged (Supplementary Figure 1, See Materials and Methods).

To avoid direct comparison to previously published work that does not account for these differences, we have omitted this statement from the current version of the manuscript.

- As outlined by the authors themselves at the conclusion of the discussion, the primary issue and limitations of this article are as follows. Due to the absence of certain samples (Fathers of P2 and S2 ; Father of N2.1, N2.2, N2.3, N2.4 and N2.5), a comprehensive analysis of the two families

cannot be conducted. Further families with POT1 mutations and age-matched controls would be required to consolidate the observations.

We agree with the reviewer that additional samples are required to support our hypotheses. To address these weaknesses, we have added an additional family trio (now Family 2). In addition, we successfully acquired and analyzed DNA from the father of the nephews in Family 3 (formerly Family 2).

- The three controls used are adults (the youngest is 38 years old, whereas most individuals tested who carry POT1 mutations are younger). Since, as demonstrated by the authors in a previous article using the same technique (Sanchez et al., Nat Comm 2024), telomere length is highly dependent on age, age-matched controls would be necessary.

We agree with the reviewer that absolute telomere length comparisons against the three controls are not informative, as the substantial inter-individual variability in telomere length limits the interpretability of such analyses even after age correction. We had explicitly stated this limitation in the manuscript and emphasized that our rank-order–based approach was developed to address this challenge (See section "The longest telomeres of children with POT1 mutations are preferentially inherited from their carrier parent"). In retrospect, we should have refrained from making absolute telomere length comparisons across families in Figure 1. Rather Controls A-C serve as quality control samples for comparing nanopore-derived TLs against TRF-measured TLs (Figure 1B, Supplemental Figure 2). As such, we have removed these comparisons from the revised manuscript. Within families, we now limit absolute telomere length comparisons to individuals of closely matched age, specifically the nephews in Family 3 and the proband and cousin in Family 2.

We have also expanded our discussion on the inter-patient variability as follows: "Notably, the bulk telomere lengths of 9 POT1 mutation carriers across 3 families, as well as 2 unrelated carriers, ranged widely, from a median length of 4.2 kb to 9.0 kb (Figure 1B). This variability is consistent with previous reports showing that not all POT1 mutation carriers exhibit elongated telomeres and likely reflects differences in allele penetrance, age, disease state, and other contributors to telomere length heterogeneity. These findings highlight the challenges of interpreting average telomere length measurements alone."

- The authors write « On average, probands displayed median telomere lengths ~1.9 kb longer than our three controls (age 56, 48, and 38)… ; ». Nevertheless, this observation is not pertinent, given that the probands P1 and P2 were analyzed at ages 5/6 and 12/17, respectively, whereas the control group comprised individuals between the ages of 38 and 56. This comparison is meaningless.

Please see our response to the comment above.

- Samples P2a and P2b are derived from bone marrow, while all other samples analyzed in this study are from blood. It is important to note that the authors have previously demonstrated that the telomere length in bone marrow cells was significantly higher than that of blood cells in the same individual (Fig3a in Sanchez et al., Nat Comm 2024). Therefore, it can be concluded that including these two samples in the comparative study would not be rigorous. P2 underwent a bone marrow transplant, which explains why blood is not available, but comparing bone marrow and blood samples from different individuals does not seem appropriate to me.

We appreciate the reviewer raising this concern. The differences in sample composition were unfortunately due a few constraints: the current study is relied on retrospective identification of POT1-carrrier families from banked samples. As Proband 3 (formerly Proband 2) had already passed at the time of sample collection for the rest of his family, we are unable to collect any additional material and, as mentioned by the reviewer, Proband 3 also underwent a bone marrow transplant. We agree with the reviewer that sample composition likely affects interpretation of absolute telomere length differences between Proband 3 and his Sister, since it is well established that peripheral blood telomeres can be up to several hundred bp shorter in peripheral blood than bone marrow (Van Ziffle, Baerlocher, and Lansdorp, 2003, Stem Cells). However, the magnitude of this difference is likely insignificant compared to the differences in age and especially cancer presentation/treatment between these two individuals.

These differences are explicitly discussed in the text: “the proband had very long telomeres in bone marrow samples that were free of cancer cells at the time of collection (median 8.5 kb), whereas his sister had significantly shorter telomeres (median 4.3 kb) in a peripheral blood sample obtained after her CLL diagnosis (Figure 1B). These differences underscore the challenges of interpreting absolute telomere length across individuals with distinct disease courses, age, treatments, and cellular compositions.”

Therefore, we include the two samples to specifically highlight that two individuals sharing the same germline variant can vary drastically in measured telomere length based on these factors.

- The authors write « 7 of 11 POT1 carriers had a significantly enriched fraction of long telomeres (>10 kb) compared to controls ». However, there are 9 POT1 carriers analyzed, not 11. Duplicate from the same individual cannot be considered as independent POT1 carriers.

Thank you for identifying this typo. The sentence has been corrected to “Notably, the bulk telomere lengths of 9 POT1 mutation carriers across 3 families, as well as 2 unrelated carriers, ranged widely, from a median length of 4.2 kb to 9.0 kb (Figure 1B).”

- Again, 7 of the 9 POT1 carriers are below age 34 while the youngest control is 38. Age-matched controls are necessary to reliably analyze interindividual variation of telomere length.

Please see above

Minor :

- To strengthen this study, it would be interesting to test the telomere length of individuals' sperm (where possible). This could help to verify that « the germline elongation « scrambles » the original telomere length order in the carrier » as proposed by the authors.

We agree with the reviewer that analyzing sperm using our sequencing approach across generations could provide important additional insights. However, we feel that these experiments are beyond the scope of this initial report, and this type of sample collection is not permitted under our current protocols. To strengthen our conclusions regarding germline telomere inheritance and the progressive scrambling of inheritance patterns caused by POT1 mutations, we now include an additional family (new Family 2) in the revised manuscript.

- It is written that « Proband samples from Families 1 and 2 were sequenced twice from independent clinical samples collected 5 years apart » however in Suppl Table 1. P1a and P1b samples are collected at age 5 and 6, respectively.

Thank you, the typo has been removed from the section “Telomere length profiling of POT1 families by Nanopore sequencing” and reflected correctly in the figure legend for Figure 1a to “twice at ages 5 and 6 and P3 sample was drawn twice at ages 12 and 17”.

- Figure 2C : The authors state that the TVR is shared by six samples. However, the profiles of samples P2a and P2b appear to differ from those of the other samples. How would the authors explain this?

We appreciate the reviewer raising this point. The TVR is highly variable between individuals because it is a region of frequent sequence changes, commonly expansion/contraction of individual repeat regions. Our use of a Levenshtein ratio of >0.85 was used to correlate TVR sequences to both account for sequencing errors introduced by Nanopre, but also to enable aggregation of alleles that have undergone minor changes in repeat composition. We found this example particularly exciting because it is detected in both Proband samples, suggesting that it is a true germline TVR variation, rather than an artifact from sequencing. We had originally omitted this discussion from our manuscript as our sample size is substantially underpowered to identify the frequency of such TVR changes, but have now noted the difference in our discussion of figure 2: “This method is also robust to minor changes in TVR sequence due to germline expansion/contraction of repeat sequences, a common TVR alteration which contributes to its extensive populational polymorphism (Figure 2D)”

- The telomere length in P1b is strongly reduced as compare to P1a while there is only 1 year of difference between the two samples. A previous study has showed that POT1 carriers have a slower rate or telomere shortening that healthy donors and non-carriers in blood cells over time (DeBoy et al., NEJM 2023). How the authors can explain this difference between the two samples P1a and P1b?

We appreciate the reviewer noting this difference. This difference was preserved across two independent sequencing events, although rank-ordering of alleles was preserved (Supplemental Figures 3 and 5). Therefore, we believe this is a biological effect rather than a technical artifact.

Proband 1 was very young at the time of sample collection (ages 5 and 6, respectively) and the most rapid changes in peripheral blood telomere length occur early in life. These differences in telomere length between the two samples could be attributable to the proliferative demand required by the child’s growth at this age, and/or the treatment required for their ependymoma. Because POT1 mutations are hugely variable in penetrance and activity, we cannot comment as to whether this rate of attrition is faster than expected without additional data.

- How to explain that M2 and S2 who are both POT1 mutation carriers have so short telomeres?

Sister 2 (now Sister 3) had active CLL at the time of sample collection. Telomere shortening is a known effect of CLL as proliferative demand outpaces the rate of compensatory telomere elongation. We have expanded our discussion on this in the section entitled “Short telomeres remain functionally relevant in a cancer-context-dependent manner.”

For the Mother, we can only speculate without additional data. It is important to note that POT1-mutation mediated telomere elongation can only occur in a telomerase-positive context. Under this context, telomeres may elongate in four important contexts: in the germline, in early development, in select telomerase-positive tissues in the adult, and in cancers where telomerase has been re-activated. In the case of the Mother, we refrain from discussing her telomere length in detail, because we do not know whether she inherited the mutation, or whether it arose de novo. The only observation we can make is that the telomere-elongating effects of the POT1 mutation were not sufficient to maintain long telomeres in the peripheral blood into her 60s. We have adjusted the text to reflect that POT1 mutation carriers across families show substantial variation in telomere length in the section entitled “Telomere length profiling of POT1 families by Nanopore sequencing”

- The analysis of telomere length in P4 is not relevant as there are not age-matched controls.

We appreciate the reviewer’s comment. These samples were not used as controls for telomere length, but rather as sequencing controls to validate the clustering of telomeres within families. As outlined above, we now refrain entirely from making absolute telomere length comparisons across families. Within families, we only compare absolute telomere lengths between the nephews in Family 3, who are of similar age, and between the cousin and proband in Family 2, who are also within a similar age range.

- The authors mention that germline mutations in POT1 are associated with familial cancer predisposition and long telomeres. However, to be exhaustive, the authors should also mention that germline mutations in POT1 have also been identified in telomeropathies with short telomeres (Tummala et al. EMBO mol med, 2024 ; Kelich, et al. J. Exp. Med 2022) as well as in Coat plus syndrome with long telomeres (Takai et al. Genes and Dev. 2016).

Yes, thank you for pointing this out. We added this now in the introduction alongside the suggested citations in the Introduction on page 3.

- The ages indicated in brackets in Figure 1 and in the Supplementary table do not correspond.

We apologize for this mistake. This has now been corrected.

- Telomere length profiles in samples N2.3 and N2.5 appear bimodal with ONT sequencing (Fig.1B) but not with TRF (Suppl. Fig1). Could you comment?

Yes, this is correct and likely originates from the known overrepresentation of long telomeres in TRFs, since long telomeres bind more probe compared to short telomeres. The increased telomere signal heterogeneity observed via TRF in Supplemental Figure 2 supports the distribution as shown in Figure 1b.

- The sentence « Despite these dramatic differences in absolute telomere length, rank-order analysis revealed a clear heriSupplementary Table ignature, with haplotypes from the POT1 carrier mother enriched in the longest third of telomeres and depleted in the shortest third in both siblings » should be edited.

Thank you for pointing this typo out, it has been fixed to state “rank-order analysis revealed a clear hereditary signature”

RESPONSE TO REVIEWERS' COMMENTS

Reviewer #1 (Remarks to the Author):

The authors have addressed my concerns and comments and I am fully supportive of publication.

We thank the reviewer for their time and insightful comments during the review process.

Reviewer #2 (Remarks to the Author):

I thank the authors for the work done during the revision.
The additions are substantial.
These were not formal requests of mine, and I want to acknowledge that the authors went well beyond what I asked.
The new data also do something more important than addressing my previous comments as they replicate the central observation independently, and this is precisely the kind of internal validation that a study based on so few families needs.
On my three original points I have little to add.
The wording on generational anticipation is now careful and, in my view, correct.
The authors no longer claim clinical evidence, they propose a mechanism, and they spell out the conditions under which such a mechanism would translate into observable anticipation. The same applies to the discussion on cross-family comparison and allele-specific penetrance, which I had raised as a clinical concern. The new paragraph acknowledges it explicitly.

One reference I would suggest adding is the recent UK clinical practice guidelines for POT1-TPDS (Hanson and colleagues, J Med Genet 2025). They are, to my knowledge, the only formal national guideline on this syndrome. They are also relevant to the authors' caveat, because the UK panel explicitly stepped back from a Li-Fraumeni-equivalent surveillance model, noting that current evidence does not support comparable lifetime cancer risk. Citing this work would strengthen the clinical contextualisation of the manuscript and would also signal to readers where the field currently stands. The findings do not, at this stage, justify any change to surveillance protocols, predictive testing thresholds, or reproductive counselling for non-carrier offspring of POT1 mutation carriers.
This is consistent with the tone the authors have already adopted and would prevent the kind of over-reading that, in my experience, tends to happen with mechanistic papers in rare cancer predisposition syndromes.

With this addition I support publication.

Reviewer #2 (Remarks on code availability):

The code is a usable resource, though I did not find any readme file with enough instructions for installing and running the application

A README.md file has been added in the "demo_data" section of Source data folder

Reviewer #3 (Remarks to the Author):

I thank the authors for their careful and substantive revision of the manuscript. I have reviewed the point-by-point response, the revised manuscript, the updated supplementary materials, and the revised figures.
Overall, the manuscript is now significantly improved in clarity, balance, and interpretive rigor.
I support publication in Nature communications, pending only minor editorial modifications.

• Line 92 typo "do"
not a typo, but the word after it is misspelt. "First, do descendants of *POT1* mutation carriers inherit aberrantly elongated telomeres?"

• Lines: 262-263. As all the nephews are healthy (lines 142-143) please change affected/unaffected by carrier/non-carrier in lines 262-263.

"unaffected" has been reworded to "non-carrier" to avoid confusion and "affected" to "carrier". Considering this, we have also changed "affected" to "carrier" in the line "In POT1 mutation positive families, where half of the telomeres of each generation derive from the..." for semantic consistency

• Many figures are of very poor quality (low definition)

This was due to an issue with embedding figures in the Word document. We will provide the full resolution vector/bitmap full resolution as PDF for the final submission.

Reviewer #3 (Remarks on code availability):

I thank the authors for their careful and substantive revision of the manuscript. I have reviewed the point-by-point response, the revised manuscript, the updated supplementary materials, and the revised figures.
Overall, the manuscript is now significantly improved in clarity, balance, and interpretive

rigor.
I support publication in Nature communications, pending only minor editorial modifications.

** See Nature Portfolio's author and referees' website at www.nature.com/authors for information about policies, services and author benefits

This email has been sent through the Springer Nature Tracking System NY-610A-NPG&MTS

Confidentiality Statement:

This e-mail is confidential and subject to copyright. Any unauthorised use or disclosure of its contents is prohibited. If you have received this email in error please notify our Manuscript Tracking System Helpdesk team at http://platformsupport.nature.com .

Details of the confidentiality and pre-publicity policy may be found here http://www.nature.com/authors/policies/confidentiality.html

Privacy Policy | Update Profile
