# Supplementary material for: Telomere length of both parents contributes to heritable POT1 cancer-predisposition syndrome
Source: Nat Commun. 2026 Sep 14;17:10797. doi: 10.1038/s41467-026-77414-2 (PMC13669393; doi:10.1038/s41467-026-77414-2)
Supplement: Supplementary file 3 [file 41467_2026_77414_MOESM3_ESM.pdf]

nature portfolio

Corresponding author(s): *Double-anonymous peer review submissions: write DAPR and your manuscript number here instead of author names.*

Last updated by author(s): *YYYY-MM-DD*

Reporting Summary

Nature Portfolio wishes to improve the reproducibility of the work that we publish. This form provides structure for consistency and transparency in reporting. For further information on Nature Portfolio policies, see our Editorial Policies and the Editorial Policy Checklist.

Statistics

For all statistical analyses, confirm that the following items are present in the figure legend, table legend, main text, or Methods section.

n/a	Confirmed	
☐	☒	The exact sample size (*n*) for each experimental group/condition, given as a discrete number and unit of measurement
☐	☒	A statement on whether measurements were taken from distinct samples or whether the same sample was measured repeatedly
☐	☒	The statistical test(s) used AND whether they are one- or two-sided<br>*Only common tests should be described solely by name; describe more complex techniques in the Methods section.*
☒	☐	A description of all covariates tested
☐	☒	A description of any assumptions or corrections, such as tests of normality and adjustment for multiple comparisons
☐	☒	A full description of the statistical parameters including central tendency (e.g. means) or other basic estimates (e.g. regression coefficient) AND variation (e.g. standard deviation) or associated estimates of uncertainty (e.g. confidence intervals)
☐	☒	For null hypothesis testing, the test statistic (e.g. *F*, *t*, *r*) with confidence intervals, effect sizes, degrees of freedom and *P* value noted<br>*Give P values as exact values whenever suitable.*
☒	☐	For Bayesian analysis, information on the choice of priors and Markov chain Monte Carlo settings
☒	☐	For hierarchical and complex designs, identification of the appropriate level for tests and full reporting of outcomes
☒	☐	Estimates of effect sizes (e.g. Cohen's *d*, Pearson's *r*), indicating how they were calculated

Our web collection on statistics for biologists contains articles on many of the points above.

Software and code

Policy information about availability of computer code

Data collection	Nanopore sequencing reads were acquired on a desktop PC equipped with a NVIDIA RTX 4090 GPU and P2Solo machine for sequencing Promethion flow cells (R10.4.1). Basecalling was performed live using MinKNOW v25.03, using basecall_model_version_id=dna_r10.4.1_e8.2_400bps_sup@v4.3.0 with the additional parameters:<br>Min Q score: 8<br>Min filter read length: 500 b<br>Run time: up to 72 hours<br><br>Data collection was additionally performed using Microsoft Excel and Graphpad PRISM 10 for Supplementary Figure 2
Data analysis	Telogator2 v2.2.0, Telometer v1.0, Python 3.12.9, custom TVR alignment code available on Github (https://github.com/Hockemeyer-Lab/PublishedCode/tree/main/POT1_Telomere_Length_Inheritance).<br><br>Graphpad PRISM 10 was used to perform simple linear regression analysis as shown in Supplementary Figure 2

For manuscripts utilizing custom algorithms or software that are central to the research but not yet described in published literature, software must be made available to editors and reviewers. We strongly encourage code deposition in a community repository (e.g. GitHub). See the Nature Portfolio guidelines for submitting code & software for further information.

Data

Policy information about availability of data

All manuscripts must include a data availability statement. This statement should provide the following information, where applicable:

- Accession codes, unique identifiers, or web links for publicly available datasets
- A description of any restrictions on data availability
- For clinical datasets or third party data, please ensure that the statement adheres to our policy

Nanopore sequencing fastq reads generated in this study are available at NCBI's Sequence Read Archive (SRA) and is available through the accession number PRJNA1420205. Remaining source data is provided with the paper.

Research involving human participants, their data, or biological material

Policy information about studies with human participants or human data. See also policy information about sex, gender (identity/presentation), and sexual orientation and race, ethnicity and racism.

Reporting on sex and gender	Sex-based analysis was only performed in the context of individual alleles inherited paternally or maternally from carrier and non-carrier parents. Due to low sample sizes, relationships are discussed individually and no aggregated sex-based conclusions are reported.
Reporting on race, ethnicity, or other socially relevant groupings	No socially constructed groupings were used. Analysis was performed on a per-family basis.
Population characteristics	Patients with POT1 tumor predisposition syndrome who underwent genetic counseling and germline genetic testing in the genetic predisposition clinic at St. Jude Children's Research Hospital were included in this study. Demographic, clinical, family history, and germline genetic test result data were collected through review of electronic medical records of patients and families affected by POT1 Tumor Predisposition. Family 2 participated in a longitudinal cohort study of individuals and families at high risk of hematologic cancer. Individual patient ages, diagnoses at time of sample collection, and treatment are outlined in the manuscript.
Recruitment	Patients were identified through retrospective analysis of samples in the St. Jude Children's Research Hospital biorepository and a longitudinal cohort study of individuals and families at high risk of hematologic cancer (ClinicalTrials.gov Identifier NCT00039676).
Ethics oversight	St. Jude Children's Research Hospital (SJCRH; IRB Number: 24-1642, Study Number: POT1) and the National Institutes of Health (ClinicalTrials.gov Identifier NCT00039676)

Note that full information on the approval of the study protocol must also be provided in the manuscript.

Field-specific reporting

Please select the one below that is the best fit for your research. If you are not sure, read the appropriate sections before making your selection.

[x] Life sciences [ ] Behavioural & social sciences [ ] Ecological, evolutionary & environmental sciences

For a reference copy of the document with all sections, see nature.com/documents/nr-reporting-summary-flat.pdf

Life sciences study design

All studies must disclose on these points even when the disclosure is negative.

Sample size	Sample size was chosen based on the availability of banked DNA from families with POT1 pathogenic variants
Data exclusions	One sample with insufficient DNA quantity or quality for Nanopore sequencing was excluded from the analysis.
Replication	Repeatability was tested by comparison of sequencing rounds of the same samples and between DNA samples for the two individuals (Proband 1 and Proband 3) who had multiple time points collected.
Randomization	Samples were not randomized as no treatments were performed. Sample inclusion requires a DNA sample from an individual with a POT1 pathogenic variant or direct relation to an individual with a pathogenic POT1 variant.
Blinding	Blinding was not possible as direct comparisons between parents/children and between siblings were critical relationships tested. TVR haplotype clustering was performed algorithmically across all samples, agnostic to family relationships.

Reporting for specific materials, systems and methods

We require information from authors about some types of materials, experimental systems and methods used in many studies. Here, indicate whether each material, system or method listed is relevant to your study. If you are not sure if a list item applies to your research, read the appropriate section before selecting a response.

Materials & experimental systems

n/a	Involved in the study
☒	☐ Antibodies
☒	☐ Eukaryotic cell lines
☒	☐ Palaeontology and archaeology
☒	☐ Animals and other organisms
☐	☒ Clinical data
☒	☐ Dual use research of concern
☒	☐ Plants

Methods

n/a	Involved in the study
☒	☐ ChIP-seq
☒	☐ Flow cytometry
☒	☐ MRI-based neuroimaging

Clinical data

Policy information about clinical studies

All manuscripts should comply with the ICMJE guidelines for publication of clinical research and a completed CONSORT checklist must be included with all submissions.

Clinical trial registration: *Provide the trial registration number from ClinicalTrials.gov or an equivalent agency.*

Study protocol: *Note where the full trial protocol can be accessed OR if not available, explain why.*

Data collection: *Describe the settings and locales of data collection, noting the time periods of recruitment and data collection.*

Outcomes: *Describe how you pre-defined primary and secondary outcome measures and how you assessed these measures.*

Plants

Seed stocks: *Report on the source of all seed stocks or other plant material used. If applicable, state the seed stock centre and catalogue number. If plant specimens were collected from the field, describe the collection location, date and sampling procedures.*

Novel plant genotypes: *Describe the methods by which all novel plant genotypes were produced. This includes those generated by transgenic approaches, gene editing, chemical/radiation-based mutagenesis and hybridization. For transgenic lines, describe the transformation method, the number of independent lines analyzed and the generation upon which experiments were performed. For gene-edited lines, describe the editor used, the endogenous sequence targeted for editing, the targeting guide RNA sequence (if applicable) and how the editor was applied.*

Authentication: *Describe any authentication procedures for each seed stock used or novel genotype generated. Describe any experiments used to assess the effect of a mutation and, where applicable, how potential secondary effects (e.g. second site T-DNA insertions, mosiacism, off-target gene editing) were examined.*
