# Supplementary material for: Telomere length of both parents contributes to heritable POT1 cancer-predisposition syndrome
Source: Nat Commun. 2026 Sep 14;17:10797. doi: 10.1038/s41467-026-77414-2 (PMC13669393; doi:10.1038/s41467-026-77414-2)
Supplement: Supplementary file 4 [file 41467_2026_77414_MOESM4_ESM.pdf]

Description of Additional Supplementary Files

File: Supplementary Data 1
Description: PatientKey.xlsx file used for Jupyter/python cross-sample aggregation analysis scripts for the manuscript. Scripts are publicly available on https://github.com/Hockemeyer-Lab/PublishedCode/tree/main/POT1_Telomere_Length_Inheritance

File: Supplementary Data 2
Description: Excel sheet of line plot densitometry values obtained via FIJI – "Plot Profile" tool and calculations performed for determining mean TRF telomere length as shown in Supplementary Figure 2

File: Supplementary Data 3
Results of telomere TVR cluster correlation, showing the consensus TVR sequence and mean levenshtein ratio for cluster correlation as well as individual sample measurements. Nan values indicate that a given TVR cluster was not identified within that sample.
